# Understanding and predicting binding between human leukocyte antigens (HLAs) and peptides by network analysis

**DOI:** 10.1186/1471-2105-16-S13-S9

**Published:** 2015-09-25

**Authors:** Heng Luo, Hao Ye, Hui Wen Ng, Leming Shi, Weida Tong, William Mattes, Donna Mendrick, Huixiao Hong

**Affiliations:** 1 National Center for Toxicological Research, U.S. Food and Drug Administration, 3900 NCTR Rd, Jefferson, AR 72079, USA; 2 University of Arkansas at Little Rock/University of Arkansas for Medical Sciences Bioinformatics Graduate Program, 2801 S University Ave, Little Rock, Arkansas, AR 72204, USA; 3 Center for Pharmacogenomics, School of Pharmacy, Fudan University, 826 Zhangheng Rd, Shanghai, 201203, China

**Keywords:** HLA, peptide, network, Nebula, binding, module, prediction, MHC

## Abstract

**Background:**

As the major histocompatibility complex (MHC), human leukocyte antigens (HLAs) are one of the most polymorphic genes in humans. Patients carrying certain HLA alleles may develop adverse drug reactions (ADRs) after taking specific drugs. Peptides play an important role in HLA related ADRs as they are the necessary co-binders of HLAs with drugs. Many experimental data have been generated for understanding HLA-peptide binding. However, efficiently utilizing the data for understanding and accurately predicting HLA-peptide binding is challenging. Therefore, we developed a network analysis based method to understand and predict HLA-peptide binding.

**Methods:**

Qualitative Class I HLA-peptide binding data were harvested and prepared from four major databases. An HLA-peptide binding network was constructed from this dataset and modules were identified by the fast greedy modularity optimization algorithm. To examine the significance of signals in the yielded models, the modularity was compared with the modularity values generated from 1,000 random networks. The peptides and HLAs in the modules were characterized by similarity analysis. The neighbor-edges based and unbiased leverage algorithm (Nebula) was developed for predicting HLA-peptide binding. Leave-one-out (LOO) validations and two-fold cross-validations were conducted to evaluate the performance of Nebula using the constructed HLA-peptide binding network.

**Results:**

Nine modules were identified from analyzing the HLA-peptide binding network with a highest modularity compared to all the random networks. Peptide length and functional side chains of amino acids at certain positions of the peptides were different among the modules. HLA sequences were module dependent to some extent. Nebula archived an overall prediction accuracy of 0.816 in the LOO validations and average accuracy of 0.795 in the two-fold cross-validations and outperformed the method reported in the literature.

**Conclusions:**

Network analysis is a useful approach for analyzing large and sparse datasets such as the HLA-peptide binding dataset. The modules identified from the network analysis clustered peptides and HLAs with similar sequences and properties of amino acids. Nebula performed well in the predictions of HLA-peptide binding. We demonstrated that network analysis coupled with Nebula is an efficient approach to understand and predict HLA-peptide binding interactions and thus, could further our understanding of ADRs.

## Background

As the major histocompatibility complex (MHC) in humans, human leukocyte antigens (HLAs) are important immunologic proteins found on the surface of somatic cells [1]. They can present antigenic peptides from the infectious agents to T-cells to induce immune responses [2-5]. People of different ethnicities or from different regions may carry distinct HLA variations or alleles [6,7]. According to IMGT/HLA database [6] by Mar 15, 2015, there are more than 12,000 alleles identified for HLAs, making the HLAs as one of the most polymorphic genes in humans. HLA genes contain multiple loci including A-G. The HLA D locus is classified as Class II and the rest are categorized as Class I due to their differences of responding T-cells and functions [2-5]. Since the binding grooves of Class I HLAs are determined by one single chain and there are a lot of peptide binding data and three-dimensional structures available [8,9], we selected Class I HLAs in this study to demonstrate the applicability of network analysis to predict HLA-peptide binding.

Patients carrying certain HLA alleles are more likely to develop adverse drug reactions (ADRs) after taking specific drugs. Drug-HLA associations have been identified between abacavir and HLA-B*57:01 [10-12], flucloxacillin and HLA-B*57:01 [13], and carbamazepine and HLA-B*15:02 [14], etc. Several mechanisms have been proposed to understand the HLA related ADRs, including the hapten concept, the super-antigen model, the p.i. concept, the altered repertoire model and the danger hypothesis [15-18]. In all the hypotheses except the danger hypothesis, the HLAs on the surface of antigen-presenting cells (APCs) present peptides to T-cell receptors (TCRs) on the surfaces of T-cells and the drug molecules interfere with the system through covalent binding to the peptides, instable interaction, or insertion into the binding grooves of HLAs. Ultimately, it is beneficial to predict ADR occurrences of drugs before patients take the drugs. However, the mechanisms for ADRs are complicated and each of the players in the system has a large number of variations in their structures, making it very challenging to study HLA related ADRs. Our previous molecular modeling study showed the drug-HLA binding prediction was improved when the binding peptide was incorporated in the modeling system [19]. Therefore, better understanding and accurately predicting HLA-peptide binding could facilitate predicting ADRs related to genetic predisposition.

Various machine learning models have been developed to predict HLA-peptide binding for individual HLAs [20-22]. However, lacking enough experimental HLA-peptide binding data to train machine learning models for many HLAs limits the capability of this approach. In addition, a significant part of machine learning models uses parameters that are derived from peptides with the same length but experiments showed the same HLA can bind peptides with different lengths, making predicting HLA-peptide binding using these methods very challenging. New methods for accurately predicting HLA-peptide binding that overcome the challenges of the reported machine learning models are in urgent need. Therefore, in this study we conducted network analysis to understand the binding characteristics between HLAs and peptides and developed a new method named neighbor-edges based and unbiased leverage algorithm (Nebula) to predict HLA-peptide binding.

Through analyzing the HLA-peptide binding network, we identified nine modules that are densely connected regions in the network [23]. Modularity is the measurement of goodness of a division of a network into modules [24] and was used to yield nine modules. The modularity of the real HLA-peptide binding network was compared to the modularity values yielded from random networks. Peptides and HLAs in the same modules shared similar properties. We further developed Nebula to predict HLA-peptide binding. To our best knowledge, this study is the first one to use network analysis for understanding and predicting HLA-peptide binding.

## Methods

### Study design

**Figure 1:**
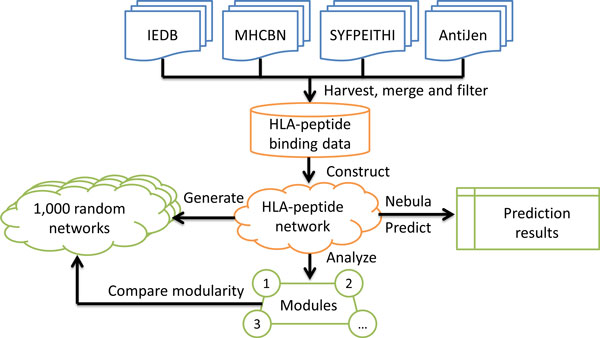
**A flowchart of this study's workflow**. Qualitative HLA-peptide binding data were harvested from IEDB, SYFPEITHI, MHCBN and AntiJen databases. The HLA-peptide binding network was generated from the binding data. The modularity analysis was then conducted on the HLA-peptide binding network and the 1,000 randomly generated networks. Finally, Nebula was used to predict HLA-peptide binding.

An overview of this study's workflow is shown in Figure 1. We first harvested qualitative Class I HLA-peptide binding data from four major databases that collected and curated HLA binding assays from the literature. The HLA-peptide binding network was then constructed from the harvested data. Thereafter, the fast greedy modularity optimization algorithm was used to identify modules. The modularity analysis on 1,000 randomly generated networks was conducted to verify that the modules yielded from the HLA-peptide binding network could not be generated by chance. Finally, we implemented Nebula to make predictions and evaluated its performance via leave-one-out (LOO) validations.

### Data preparation and network construction

Four major databases, IEDB [25], SYFPEITHI [26], MHCBN [27] and AntiJen [28], contain HLA-peptide binding data curated from the literature. IEDB, MHCBN and AntiJen provided qualitative binding categories (positive or negative), while SYFPEITHI contains all positive bindings. For IEDB data, "positive-high", "positive-intermediate" and "positive-low" were all considered as positives. For AntiJen data, the "weak binders" are considered as negatives according to the paper's description [28].

We harvested the qualitative binding data of Class I HLA-peptide binding assays from the four databases by Aug 25, 2014 and combined all data into a single dataset. The dataset contains three columns: HLA, peptide and binding category (positive or negative). For an HLA-peptide pair with multiple entries in the databases, we calculated the proportion of positives. If the proportion is larger than or equal to 0.5, then it was stored as a unique record of positive (otherwise it was labelled as a negative). We removed the peptides and HLAs that contain only one binding datum for two reasons: 1) the datum may be in low quality because only the HLA or the peptide shows binding among such a large number of peptides or HLAs; and 2) it would not be able to be predicted by Nebula because no data for the HLAs or peptides could be used for the predictions in the LOO validations. The data filtering process was run through several iterations to make sure all the peptides and HLAs had more than one binding datum. To enable the calculations in the network analysis, we used 2 and 1 to represent positive and negative, respectively. Finally, the HLA-peptide binding network was constructed using the igraph package (version 0.7.1) in R 3.1.3.

### Module identification and modularity analysis

We used the fast greedy modularity optimization algorithm by Clauset et al. [24] via the igraph package to identify modules within the HLA-peptide binding network. This algorithm is well-known for its advantage to fast detect modules from large networks [29]. To examine whether the yielded modules really have binding characteristics for HLAs and peptides or could be generated by chance, we generated 1,000 random networks using three criteria: (1) the network topology, both nodes and edges, in the random networks remain the same as in the real HLA-peptide binding network; (2) only weights (positive or negative) were randomly shuffled, while keeping the same amount of positives and negatives; and (3) the modules from random networks were generated using the same algorithm and parameters. The modularity values [24] were then compared between the real HLA-peptide binding network and its randomly permutated networks.

### Comparative analysis of modules

The modules yielded from modularity analysis of the HLA-peptide binding network were compared in terms of both the HLAs and the peptides. To compare the HLAs across modules, the available protein sequences of all the HLAs were first downloaded from the IMGT/HLA database [6]. The HLA sequences were then aligned using the MUSCLE method in MEGA 5.2.1 [30] with default parameters. Chelvanayagam [8] identified a uniform list of HLA residues that specifically interact with each amino acid (AA) position in 9-mer peptides. These residue numbers were given referring to the sequence of A*02:01 (PDB ID: 3HLA) [9]. For each AA position, we extracted the corresponding residues from all the HLA sequences and put them together as position-specific pseudo-sequences. The pairwise sequence identities of the pseudo-sequences were calculated using Clustal Omega 1.2.0 [31].

### Nebula

Nebula was developed through modification of the collaborative filtering algorithm [32] and the network-based inference (NBI) method [33,34]. The HLA-peptide binding data can be constructed into a weighted bipartite network, where an edge is drawn between HLA *h_i _*and peptide *p_x _*if there is a binding datum (positive or negative) between them. The edge weight is given by $w_{h_{i},p_{x}}$ in equation (1).

(1) $$w_{h_{i},p_{x}}=\left\{\begin{array}{c} 2,if\;positive\;binding\\ 1,if\;negative\;binding \end{array}\right)$$

If HLA *h_i _*and peptide *p_x _*do not have a binding datum, a prediction value for the edge between *h_i _*and *p_x _*can be calculated from the edges neighboring to the edge in prediction using equations (2-6).

(2) $$P_{h_{i},p_{x}}=\bar{w_{h_{i}}}+\frac{\sum_{j}\left(w_{h_{j},p_{x}}-\bar{w_{h_{i}}}\right)S_{h_{i},h_{j}}}{\sum_{j}\left|S_{h_{i},h_{j}}\right|}$$

$\bar{w_{h_{i}}}$ is the average weight of all edges that connect to HLA *h_i_, h_j _*is a HLA that connects to peptide *p_x_*, and $S_{h_{i},h_{j}}$ is the Pearson correlation coefficient between *h_i _*and *h_j _*and calculated using equation (3).

(3) $$S_{h_{i},h_{j}}=\frac{\sum_{a}\left(w_{h_{i},p_{a}}-\bar{w_{h_{i}}}\right)\left(w_{h_{j},p_{a}}-\bar{w_{h_{j}}}\right)}{\sqrt{\sum_{a}{\left(w_{h_{i},p_{a}}-\bar{w_{h_{i}}}\right)}^{2}\sum_{a}{\left(w_{h_{j},p_{a}}-\bar{w_{h_{j}}}\right)}^{2}}}$$

*p_a _*indicates a peptide that connects to both *h_i _*and *h_j_*. When *h_i _*and *h_j _*do not share a connected peptide, $S_{h_{i},h_{j}}=0$. Likewise, $P_{p_{x},h_{i}}$ can be calculated using equation (4).

(4) $$P_{p_{x},h_{i}}=\bar{w_{p_{x}}}+\frac{\sum_{k}\left(w_{p_{k},h_{i}}-\bar{w_{p_{k}}}\right)S_{p_{x},p_{k}}}{\sum_{k}\left|S_{p_{x},p_{k}}\right|}$$

$\bar{w_{p_{x}}}$ is the average weight of all edges that connect to peptide *p_x_, p_k _*is a peptide that connects to HLA *h_i_*, and $S_{p_{x},p_{k}}$ is the Pearson correlation coefficient between *p_x _*and *p_k _*and calculated using equation (5).

(5) $$S_{p_{x},p_{k}}=\frac{\sum_{a}\left(w_{p_{x},h_{a}}-\bar{w_{p_{x}}}\right)\left(w_{p_{k},h_{a}}-\bar{w_{p_{k}}}\right)}{\sqrt{\sum_{a}{\left(w_{p_{x},h_{a}}-\bar{w_{p_{x}}}\right)}^{2}\sum_{a}{\left(w_{p_{k},h_{a}}-\bar{w_{p_{k}}}\right)}^{2}}}$$

*h_a _*is a HLA that connects to both *p_x _*and *p_k_*. When *p_x _*and *p_k _*do not share a connected HLA, $S_{p_{x},p_{k}}=0$.

Nebula treats the contributions from the edges connected to the two nodes of the edge equally in prediction, that is $P_{h_{i},p_{x}}$ and $P_{p_{x},h_{i}}$. Therefore, the final prediction value between HLA *h_i _*and peptide *p_x _*as *F(h_i_,p_x_) *is calculated using equation (6).

(6) $$F\left(h_{i},p_{x}\right)=\frac{P_{h_{i},p_{x}}+P_{p_{x},h_{i}}}{2}$$

*F(h_i_,p_x_) *is a continuous value which is converted into a categorical prediction value *C(h_i_,p_x_) *in Nebula using the unbiased leverage (UL) as presented by equation (7). Since we assigned the weights for positive binding as 2 and negative as 1, the *UL *was set to be 1.5.

(7) $$C\left(h_{i},p_{x}\right)=\left\{\begin{array}{c} positive,\;if\;F\left(h_{i},p_{x}\right)\geq UL\\ negative,\;if\;F\left(h_{i},p_{x}\right)<UL \end{array}\right)$$

### Evaluation of Nebula performance

To evaluate the performance of Nebula, we used LOO validations. Each of the edges was taken out one at a time from the HLA-peptide binding network, and the remaining network was used to predict the weight of the taken-away edge. A receiver operating characteristic (ROC) curve was generated using the continuous final prediction values *F(h_i_,p_x_) *against the binding labels using AUC package in R (version 0.3.0). Sensitivity, specificity and accuracy were calculated by comparing the categorical prediction values *C(h_i_,p_x_) *against the labels determined from HLA-peptide binding assays. We did a similar evaluation for NBI method [34] as a comparison. The author of NBI, Dr. Feixiong Cheng, provided the NBI codes to us.

Two-fold cross-validations were also conducted to eliminate potential over-fitting from the LOO validations. Each time the entire HLA-peptide binding network was randomly divided into two even portions and each portion was used to predict HLA-peptide binding in the other portion. We ran 100 iterations and calculated the sensitivity, specificity, accuracy and area under the ROC curve (AUC) values to measure the performance of Nebula.

## Results and discussion

### Modularity analysis

**Figure 2:**
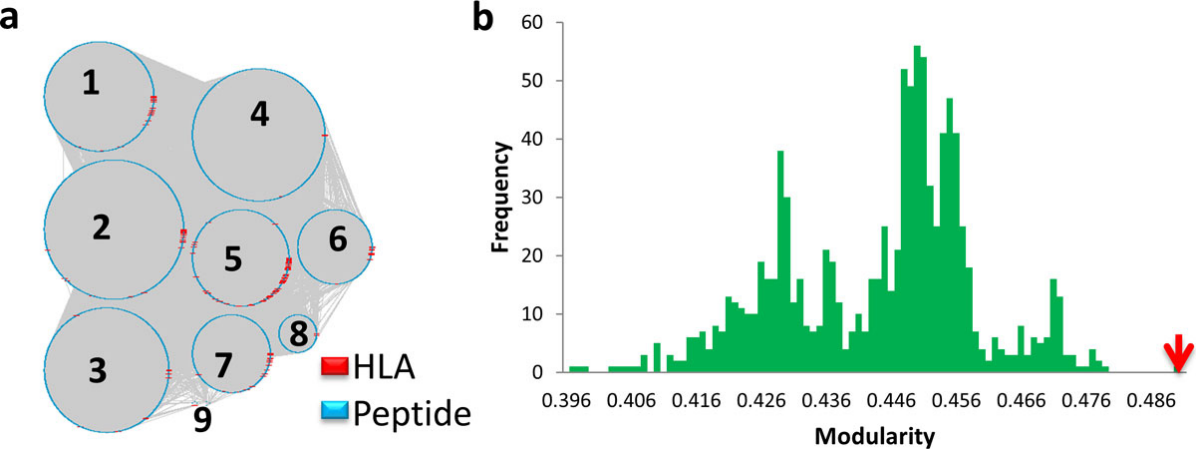
**Results of modularity analysis**. Nine modules were generated from the HLA-peptide binding network and plotted via Cytoscape 3.2.0 (**a**). The HLAs are shown in red, peptides in cyan and edges in grey. Modularity values of 1,000 randomly permutated networks are given in the histogram (**b**). As a comparison, the modularity value yield from the real HLA-peptide binding network is indicated by the red arrow.

After data pre-processing, we obtained 118,959 binding data points (39.6% positives and 60.4% negatives) between 18,630 peptides and 211 Class I HLAs for network construction and modularity analysis (Supplementary Table S1 in Additional file 1). Nine modules were identified from the HLA-peptide binding network using the fast greedy modularity optimization algorithm as shown in Figure 2a. A modularity value of 0.489 was found. The calculated results of the peptides and HLAs in the nine modules are given in Table 1. The sequences of the peptides and HLAs in the nine modules are listed in Supplementary Table S2 and S3, respectively, in Additional file 1.

**Table 1:** Statistics of peptides and HLAs in the nine modules

Module	Peptides							HLA count
	**7-mer**	**8-mer**	**9-mer**	**10-mer**	**11-mer**	**>11-mer**	**Total**	

1	0.0%	3.2%	**15.8%**	**11.0%**	2.5%	0.3%	2578	25
2	0.0%	7.3%	**30.2%**	5.3%	7.8%	0.3%	4175	43
3	**14.3%**	0.5%	**14.7%**	**29.7%**	4.6%	0.3%	3356	12
4	0.0%	**21.0%**	**17.6%**	**29.8%**	9.5%	0.5%	3797	7
5	**85.7%**	**35.6%**	8.6%	6.3%	**29.7%**	91.2%	1936	86
6	0.0%	**18.7%**	5.5%	7.6%	**20.1%**	2.3%	1187	13
7	0.0%	**13.7%**	5.3%	**10.0%**	**25.8%**	5.3%	1295	22
8	0.0%	0.0%	2.2%	0.4%	0.0%	0.0%	303	2
9	0.0%	0.0%	0.0%	0.0%	0.0%	0.0%	3	1
Total	7	219	12819	4906	283	396	18630	211

Peptide length distributions are presented as percentages for each model. Percentages equal to or larger than 10% are highlighed in bold.

Using the same modularity analysis algorithm, we analyzed the 1,000 randomly generated networks. The 1,000 modularity values were plotted as a histogram shown in Figure 2b. All the 1,000 modularity values are lower than the modularity yielded from the real HLA-peptide binding network (the red arrow in Figure 2b), indicating the nine modules harvested by the algorithm is not likely some result obtained by chance. In order to discover potential signals buried in the nine modules, we analyzed the peptide and HLA properties across the modules.

In this modularity comparison, we used very strict criteria to generate the random networks not even altering the topology of the original network. Another way is to generate random networks by reconnecting edges while keeping the same amount of nodes, positive and negative edges, which resulted in modularity values with even larger differences (data not shown).

### Peptide analysis across the modules

The distributions of peptides and HLAs in the nine modules are listed in Table 1. For peptides with a specific length such as 8-mers, the peptide distribution across the modules is also shown in Table 1 by column with the column sum equal to 100%. Using 10% as a cutoff, we found 8-mer and 11-mer peptides are more likely to appear in modules 4-7, while 9-mers and 10-mers majorly exist in modules 1-4. Modules 1-3 and 5-7 show a higher specificity on peptide lengths while module 4 is a mixture of 8-mers, 9-mers and 11-mers. The results indicate peptides in different modules may have different binding interactions with HLAs.

**Figure 3:**
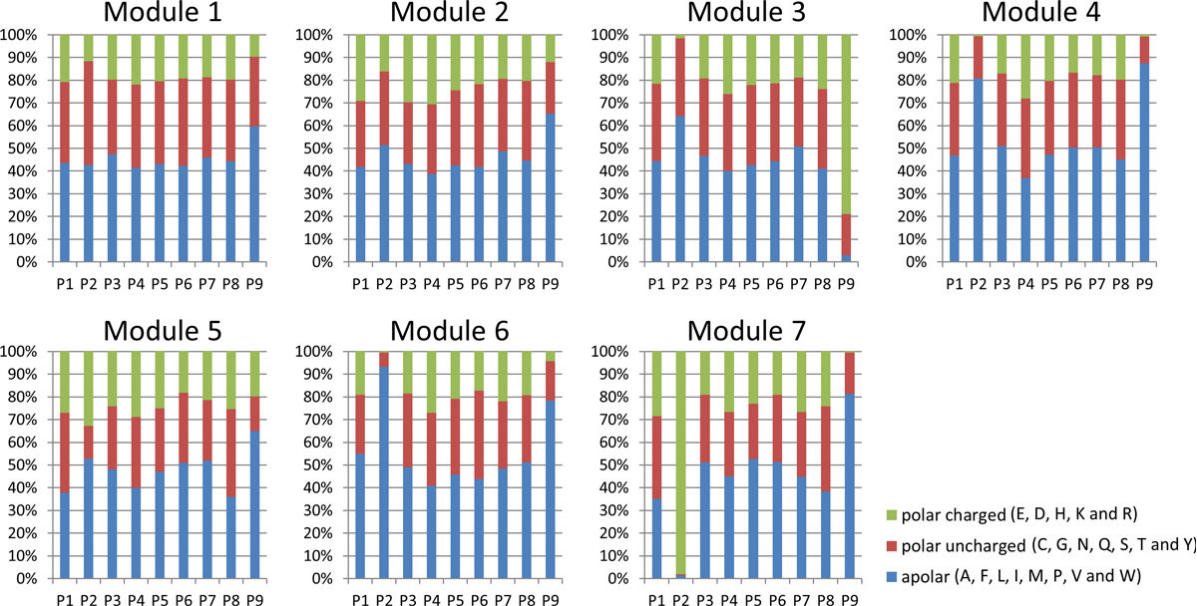
**The distribution of amino acid residues by position from 9-mer peptides in each module**. Each bar chart shows the 9-mer peptides from a module indicated at the top of the bar chart. The x-axis marks P1 to P9 represent positions 1 to 9 in 9-mers. At each position, 20 possible amino acid residues were categoried into three groups: polar charged (green), polar uncharged (red) and apolar (blue). The percentages of the three groups are shown at each position.

Modules 1-7 are the major modules that contain more than 1,000 peptides and 9-mers are the majority of peptides (68.8%). We further analyzed 9-mer peptides across these modules to explore HLA-peptide binding characteristics of the modules. According to Hong et al. [35], the common 20 amino acids (AAs) can be categorized into 3 groups: (1) polar charged (Arg, Asp, Glu, His and Lys), (2) polar uncharged (Asn, Cys, Gln, Gly, Ser, Thr and Tyr), and (3) apolar (Ala, Ile, Leu, Met, Phe, Pro, Trp and Val). For each module, we categorized the AA residues within the 9-mer peptides into the three groups from Position 1 to 9. The result is shown in Figure 3.

We found the distributions of AA residue categories are similar across different modules for most positions. However, two positions showed very distinct characteristics across modules. For position 2 (P2), while module 4 contains 80.8% apolar AA residues and module 6 includes 93.2% apolar AA residues, module 7 has a dominant proportion of polar charged AA residues (up to 98.1%). For the last position (PΩ, or P9 for 9-mer peptides), while module 3 showed a majority of polar charged AA residues (79.0%), modules 4, 6 and 7 had 87.5%, 78.4% and 81.4% of apolar AA residues, respectively. Detailed information regarding AA residue distributions at Position 2 and 9 in each module compared to their overall distributions among all nine modules is attached in Supplementary Table S4 in Additional file 1. Similar results were also observed for other lengths of peptides (results not shown). Therefore, we found differences in peptide lengths and properties across the modules.

It has been reported that position 2 (P2) and, especially the last position (PΩ), of peptides have critical effects on drug binding inside the HLA binding grooves which may affect the occurrence of HLA related ADRs [16,36]. Our network analysis differentiated peptides with specific amino acid properties into different modules and clustered those similar ones together in certain modules, which could be useful to further our understanding of HLA related ADR mechanisms.

### HLA sequence analysis across the modules

**Figure 4:**
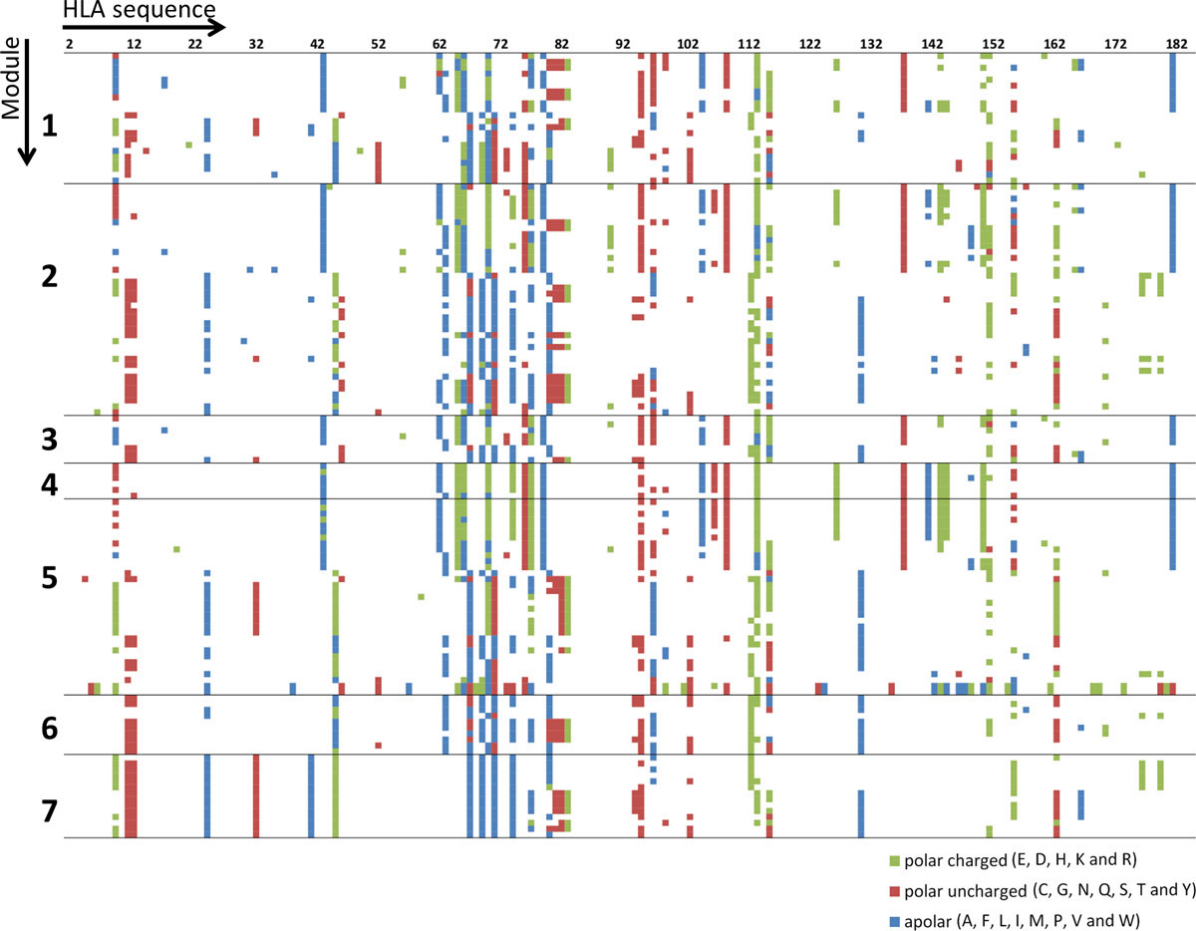
**The HLA sequence difference across the modules colored by residue category**. HLA sequences from seven major modules are shown. Each line is an HLA partial sequence (residues 2 to 182 on Chain A) of the peptide-binding region. Each column represents a residue at the same position across all the HLAs. For each column, if a residue has their highest frequency within the column, it is not shown (white color). Otherwise, it is colored according to the three categories as explained in the legend to Figure 3.

Since Class I HLA alleles are highly similar, the sequences of their peptide-binding regions (residues 2 to 182, Chain A) aligned well without a single gap. These partial HLA sequences are given in Supplementary Table S3 in Additional file 1. To assess the sequential differences of HLAs across the modules, we plotted the available sequences in Figure 4 to highlight the residues that are different from the most frequent residue for each position. The different residues are colored according to the three AA categories mentioned above.

We observed some HLA sequence differences in the modules at certain sequence positions. For example, in module 7, the residues at position 24 and 67 are apolar and the residues at position 45 are polar charged, which showed a uniformity and difference against the rest of the modules. Since these three HLA residues are reported to interact with position 2 of 9-mer peptides [8], combining the peptide sequence analysis results that indicated the position 2 of 9-mer peptides in module 7 is dominant by polar charged residues (98.1%), we think the peptides and HLAs in the same modules are concordant to form specific binding patterns.

We also analyzed the identities of HLA pseudo-sequences that specifically interact with each AA position of 9-mer peptides, and the results for positions 2 and 9 are given in Supplementary Table S5 in Additional file 1. For positions 2 and 9, we found the pseudo-sequence identities within the modules are generally significantly higher (*p *< 0.05) than those between modules. Especially, for position 2 pseudo-sequences, module 7 had the highest average identity within the module and lowest average identity between the modules.

In summary, this study revealed that not only the peptides, but also HLA sequences showed more similarities and concordant properties within the modules than between the modules. Modularity analysis of the HLA-peptide binding network is helpful to understand HLA-peptide binding interactions that, in turn, could facilitate understanding of HLAs related ADRs.

### Validations for Nebula

**Figure 5:**
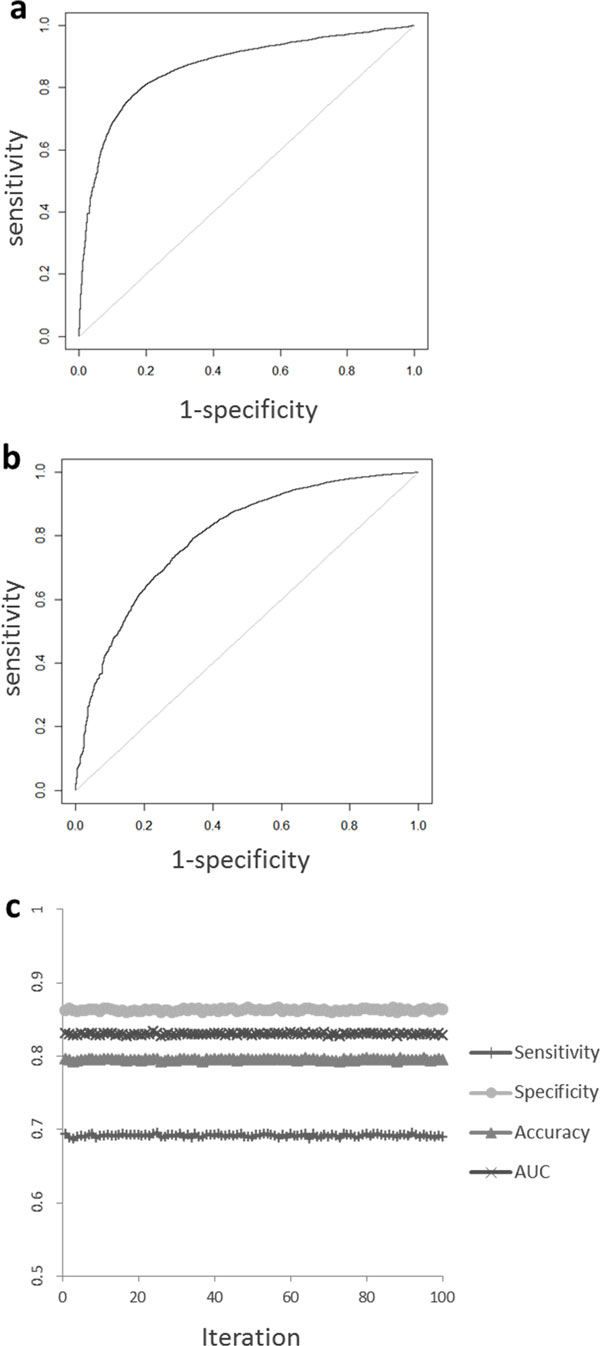
**HLA-peptide binding prediction performance**. The comparison of ROC curves of leave-one-out validations were plotted for Nebula (**a**) and NBI (**b**). The sensitivity, specificity, accuracy and AUC values were plotted for the 100 iterations of two-fold cross-validations (**c**).

To evaluate the performance of Nebula, LOO validations were conducted in which each of the 118,959 HLA-peptide binding data was left out for prediction by the network constructed from the rest of the 118,958 binding data points. The results were plotted as a ROC curve shown in Figure 5a. AUC was calculated to be 0.868. The sensitivity, specificity and accuracy are 70.8%, 88.7% and 81.6%, respectively.

As a comparison, the performance of NBI for predicting HLA-peptide binding in the same dataset was evaluated using the same LOO validations. The results were given in Figure 5b, with a lower AUC of 0.799. The sensitivity, specificity and accuracy are 33.2%, 94.4% and 68.1%, respectively. The results indicated that Nebula generally outperformed NBI and holds a promising application in analyzing big and sparse datasets such the HLA-peptide binding dataset used in this study.

In order to reduce the potential over-fitting from the LOO validations, we conducted 100 iterations of two-fold cross-validations for Nebula. The results were shown in Figure 5c. The sensitivity, specificity, accuracy and AUC values are 69.2% ± 0.2%, 86.3% ± 0.2%, 79.5% ± 0.1% and 0.830 ± 0.001, respectively, slight lower than the LOO validations as expected, indicating over-fitting is not a big concern for Nebula.

Machine learning methods such as artificial neuron network (ANN) [37] and support vector machine (SVM) [38] were used for HLA-peptide binding predictions. However, most conventional machine learning methods have been applied for a limited number of HLAs and peptides of specific lengths because they require a large enough amount of data to train a reliable prediction model. Moreover, the independent variables used in most reported HLA-peptide binding prediction models were derived from peptides with a fixed length unless an extra process [39] was implemented to process peptides with different lengths. As demonstrated by the results, Nebula not only achieved good prediction accuracy, but also does not require a large amount of experimental data for an HLA allele or a fixed length for peptides.

## Conclusions

We identified nine modules in the HLA-peptide binding network using the fast greedy modularity optimization algorithm. The modules showed distinct distributions and properties for both peptides and HLAs across the modules, indicating network analysis is a promising approach to understand structures and characteristics of big and sparse data. We developed Nebula for prediction based on network analysis and used HLA-peptide binding dataset as a case study to demonstrate it is reliable and practicable for big data analysis. Our results suggest that the network analysis methods such as Nebula are applicable and effective to interpret and predict large and sparse datasets such as the HLA-peptide binding dataset used in this study. We showed such methods could accurately predict HLA-peptide binding that, in turn, could improve predictions of HLA related ADRs to better implement precision medicine.

## List of abbreviations used

AA: amino acids; ADR: adverse drug reaction; ANN: artificial neuron network; AUC: area under the ROC curve; HLA: human leukocyte antigen; IEDB: Immune Epitope Database; LOO: leave-one-out; MHC: major histocompatibility complex; NBI: network-based inference; Nebula: neighbor-edges based and unbiased leverage algorithm; P2: Position 2; PDB: Protein Data Bank; PΩ: last position; ROC: receiver operating characteristic; SVM: support vector machine; TCR: T-cell receptor; UL: unbiased leverage.

## Competing interests

The authors declare that they have no competing interests.

## Authors' contributions

HH, DM, LS, WM and WT designed and led the project. HL collected the data. HL and HY implemented the methods. HL, HY, HN and HH discussed the data analysis and the results. HL, HH and DM wrote the manuscript.

## Disclaimer

The findings and conclusions in this article have not been formally disseminated by the US Food and Drug Administration (FDA) and should not be construed to represent the FDA determination or policy.

## Supplementary Material

Additional File 1 **Supplementary Tables S1, S2, S3, S4 and S5**. Supplementary Table S1. The processed HLA-peptide binding data for network construction. Supplementary Table S2. The peptides in each module. Supplementary Table S3. The HLAs in each module. Supplementary Table S4. Residue distributions at Positions 2 and 9 for 9-mer peptides in seven major modules compared to their overall distributions among all nine modules. Supplementary Table S5. Pseudo-sequence identities within and between modules for Positions 2 and 9. Click here for file

## Declarations

The publication charges for this article were funded by NCTR appropriated funds.

This article has been published as part of *BMC Bioinformatics *Volume 16 Supplement 13, 2015: Proceedings of the 12th Annual MCBIOS Conference. The full contents of the supplement are available online at http://www.biomedcentral.com/bmcbioinformatics/supplements/16/S13.
